# Supplementary material for: Practical Indicators for Risk of Airborne Transmission in Shared Indoor Environments and Their Application to COVID-19 Outbreaks
Source: Environ Sci Technol. Author manuscript; Available in PMC 2024 Dec 11. (PMC7617205; doi:10.1021/acs.est.1c06531)
Supplement: Supporting information — The Supporting Information is available free of charge at https://pubs.acs.org/doi/10.1021/acs.est.1c06531. Derivation of the factor accounting for the deviation from the steady state, details of Monte Carlo uncertainty propagation for the fitting of attack rates vs Hr, evaluation of the fitting in Figure 1b, rE and rB for different vocalization and physical intensity levels, details of the conditions for Table 2, details of the setting in Figure 2, and screenshot of the COVID-19 Aerosol Transmission Estimator (PDF) [file EMS181949-supplement-Supporting_information.pdf]

Supporting Information for Peng et al., "Practical Indicators for Risk of Airborne Transmission in Shared Indoor Environments and their Application to COVID-19 Outbreaks"

Z. Peng,[1] A.L. Pineda Rojas,[2] E. Kropff,[3] W. Bahnfleth,[4] G. Buonanno,[5] S.J. Dancer,[6,7] J. Kurnitski,[8] Y. Li,[9] M.G.L.C. Loomans,[10] L.C. Marr,[11] L. Morawska,[12] W. Nazaroff,[13] C. Noakes,[14] X. Querol,[15] C. Sekhar,[16] R. Tellier,[17] T. Greenhalgh,[18] L. Bourouiba,[19] A. Boerstra,[20] J.W. Tang,[21] S.L. Miller,[22] and J.L. Jimenez[1*]

Affiliations

1. Dept. of Chemistry and CIRES; University of Colorado, Boulder, CO 80309, USA

2. CIMA, UMI-IFAECI/CNRS, FCEyN, Universidad de Buenos Aires—UBA/CONICET, Buenos Aires C1428EGA, Argentina

3. Leloir Institute—IIBBA/CONICET, CBA, Buenos Aires C1405BWE, Argentina

4. Dept. of Architectural Engineering, The Pennsylvania State University, University Park, PA 16802, USA

5. Dept. of Civil and Mechanical Engineering, University of Cassino and Southern Lazio, 03043 Cassino, Italy

6. Dept. of Microbiology, NHS Lanarkshire, Scotland G75 8RG, UK

7. School of Applied Sciences, Edinburgh Napier University, Scotland EH11 4BN, UK

8. REHVA Technology and Research Committee, Tallinn University of Technology, 19086 Tallinn, Estonia

9. Dept. of Mechanical Engineering, the University of Hong Kong, Hong Kong, China

10. Dept. of the Built Environment, Eindhoven University of Technology, Eindhoven, 5612 AZ, the Netherlands

11. Dept. of Civil and Environmental Engineering, Virginia Tech, Blacksburg, VA 24061, USA

12. International Laboratory for Air Quality and Health, Queensland University of Technology, Brisbane, QLD 4001, Australia

13. Dept. of Civil and Environmental Engineering, University of California, Berkeley, CA 94720, USA

14. School of Civil Engineering, University of Leeds, Leeds LS2 9JT, UK

15. Institute of Environmental Assessment and Water Research, IDAEA, Spanish Research Council, CSIC, 08034 Barcelona, Spain

16. Dept. of the Built Environment, National University of Singapore 117566, Singapore

17. Dept. of Medicine, McGill University and McGill University Health Centre, Montreal, QC H4A 3J1, Canada

18. Nuffield Dept. of Primary Care Health Sciences, University of Oxford, Oxford OX2 6GG, UK.

19. The Fluid Dynamics of Disease Transmission Laboratory, Massachusetts Institute of Technology, Cambridge, MA 02139, USA

20. REHVA (Federation of European Heating, Ventilation and Air Conditioning Associations), BBA Binnenmilieu, The Hague, 2501 CJ, the Netherlands

21. Dept. of Respiratory Sciences, University of Leicester, Leicester LE1 7RH, UK

22. Dept. of Mechanical Engineering, University of Colorado, Boulder, CO 80309, USA

*: corresponding author: <jose.jimenez@colorado.edu>

Supporting Information Table of Contents:

Total Pages: 21

Table of Figures

Figure S1	Predicted vs. actual attack rates of the COVID-19 outbreaks shown in Table 1	S16
Figure S2	Screenshot of the COVID-19 Aerosol Transmission Estimator	S17
Figure S3	Ratio of the average quanta concentration to that at steady state as a function of the product of total first-order quanta loss rate constant and the event duration	S18

Table of Tables

Table S1	Mathematical symbols used in this study	S4
Table S2	Relative factors of quanta emission and volumetric breathing rates for different activities	S7
Table S3	Values of the parameters used for computation of Table 2 in the main paper	S10
Table S4	Parameters for pre-pandemic use of various indoor spaces, and for possible pandemic lower-risk scenarios	S12

Table S1: mathematical symbols used in this study.

Symbol	*Physical meaning*	*Unit (dimension -less if no unit indicated)*
B	Volumetric breathing rate of a susceptible person	$m^3\ h^{-1}$
B_0	Volumetric breathing rate of a resting susceptible person	$m^3\ h^{-1}$
c	Virus concentration	quanta m^{-3}
c_{avg}	Average virus concentration in the air over the duration of the event	quanta m^{-3}
D	Duration of the event	h
E_p	SARS-CoV-2 exhalation rate by an infector	quanta h^{-1}
E_{p0}	SARS-CoV-2 exhalation rate by an infector resting and only orally breathing	quanta h^{-1}
f_e	Exhalation penetration efficiency for face covering	
f_i	Inhalation penetration efficiency for face covering	
H	Infection risk parameter, as defined in equation (11)	persons $h^2\ m^{-3}$
H’	Infection risk parameter without activity taken into account, as defined in equation (14)	persons $h^2\ m^{-3}$
H_r	Relative infection risk parameter, as defined in equation (15)	$h^2\ m^{-3}$

η_I	Probability of an occupant being an infector	
λ	First-order overall rate constant of the virus infectivity loss	h^{-1}
λ_0	Ventilation rate	h^{-1}
λ_{cle}	Virus removal rate by cleaning devices	h^{-1}
λ_{dec}	Virus infectivity decay rate	h^{-1}
λ_{dep}	Deposition rate of airborne virus-containing particles onto surfaces	h^{-1}
L	Ventilation rate per susceptible person	liter s^{-1} $person^{-1}$
N	Number of occupants	
N_i	Number of infectors	
N_{sus}	Number of susceptible persons	
N_{si}	Number of secondary infections	
n	Amount of the virus infectious doses inhaled by a susceptible person in a given indoor environment	quanta
P	Probability of infection of a susceptible person conditional on the presence of an infector	
P_a	Absolute probability of infection of a susceptible person	
r_{ss}	Ratio of the average virus concentration to that at steady state	

r_B	Relative breathing rate enhancement factor (vs. B_0) for an activity	
r_E	Relative virus exhalation rate enhancement factor (vs. E_{p0}) for an activity	
V	Indoor environment volume	m^3

Table S2: relative factors of (a) quanta emission and (b) volumetric breathing rates for different activities according to refs [1,2] and ref [3], respectively. The values of relative quanta emission rate factor for moderate exercise in (a) are interpolated as in ref [4].

(a)

Activity		Relative quanta emission rate factor
Physical intensity	Vocalization	
Resting	Oral breathing	1
	Speaking	4.7
	Loudly speaking	30.3
Standing	Oral breathing	1.2
	Speaking	5.7
	Loudly speaking	32.6
Light exercise	Oral breathing	2.8
	Speaking	13.2
	Loudly speaking	85
Moderate exercise	Oral breathing	4.3
	Speaking	20.4
	Loudly speaking	132

Heavy exercise	Oral breathing	6.8
	Speaking	31.6
	Loudly speaking	204

(b)

Age group (year)	Activity level				
	Sleep or nap	Sedentary /passive	Light intensity	Moderate intensity	High intensity
<1	0.63	0.64	1.6	2.9	5.4
1 - <2	0.94	1.0	2.5	4.4	7.9
2 - <3	0.96	1.0	2.5	4.4	8.1
3 - <6	0.90	0.94	2.3	4.4	7.7
6 - <11	0.94	1.0	2.3	4.6	8.7
11 - <16	1.0	1.1	2.7	5.2	10
16 - <21	1.0	1.1	2.5	5.4	10
21 - <31	0.90	0.88	2.5	5.4	10
31 - <41	1.0	0.89	2.5	5.6	10
41 - <51	1.0	1.0	2.7	5.8	11

51 - <61	1.1	1.0	2.7	6.0	11
61 - <71	1.1	1.0	2.5	5.4	9.8
71 - <81	1.1	1.0	2.5	5.2	9.8
≥81	1.1	1.0	2.5	5.2	10
Average	1.0	1.0	2.4	5.0	9

Table S3: values of the parameters used for computation of Table 2 in the main paper.

**Relative quanta emission factor**		
Silent	1	
Speaking	5	
Shouting, singing	30	
Heavy exercise	7	
**Relative breathing rate factor**		
Silent	1	
Speaking	1	
Shouting, singing	1	
Heavy exercise	10	
**Low occupancy**	10	persons
**High occupancy**	35	persons
**Ventilation rate**		
Outdoor and well ventilated	500	ACH
Indoor and well ventilated	6	ACH
Poorly ventilated	1	ACH
**Face coverings**		
Exhalation filtration efficiency	50%	
Inhalation filtration efficiency	30%	
**Contact time**		
Short	1	h
Long	10	h
**Effective volume**		
Indoor	300	m^3
Outdoor	300	m^3

Footnote: A rough estimate can be obtained as follows. To be comparable with the indoor volume, the outdoor volume is assumed to be the same as the indoor one (10 m x 10 m x 3 m box). The outdoor ventilation rate

corresponds to the ventilation by wind passing through a horizontal dimension of the outdoor box (10 m) at 5 km h^{-1} (~1.4 m s^{-1}, toward the low end of the monthly mean wind speed in US cities).[5] The outdoor box dimensions and wind speed are input parameters for the table in the same format in the COVID-19 Aerosol Transmission Estimator (Figure S2) for its users to more easily estimate equivalent outdoor ventilation.

Table S4: parameters for pre-pandemic use of various indoor spaces, and for possible lower-risk scenarios while COVID-19 is active (rows in gray). The predicted number of secondary cases is estimated based on the fitted trend in Figure 1b. The ventilation rates (λ_0) of the ASHRAE standard cases correspond to the minimum requirement recommended in ref [6]. The other cases are based on real-world indoor spaces or reasonable estimation. r_E and r_B are estimated mostly based on the typical values for all-age-group averages in Table S2. No additional virus-removal devices or no face covering are used in the pre-pandemic cases. Common measures for the lower-risk scenarios in this table are half occupancy, half duration, surgical mask wearing (f_e x f_i = 0.35),[4,7] and use of additional virus-removing devices (e.g. HEPA filter) with λ_{cle} = 3 h^{-1}. Two literature outbreaks in Table 1, i.e. the Guangzhou restaurant[8] and Skagit Choir[9] cases, are also shown for comparison. See footnotes for the exceptions to these descriptions.

Indoor environment type	r_E	r_B	$f_e x f_i$	D (h)	N_{sus}	V (m^3)	$\lambda_0 + \lambda_{cle}$ (h^{-1})	r_{ss}	H (persons h^2 m^{-3})	H_r (h^2 m^{-3})	Predicted number of secondary cases
ASHRAE standard cases											
Prison dayroom	2.8	2.4	1	8	300	5.0E+03	0.76	0.84	3.6E+00	1.2E-02	1.8E+01
	2.8	2.4	0.35	4	150	5.0E+03	3.8	0.93	7.0E-02	4.7E-04	3.8E-01
Middle school classroom	1	1.1[a]	1	5	20	1.7E+02	2.8	0.93	2.1E-01	1.1E-02	1.1E+00
	1	1.1[a]	0.35	2.5	10	1.7E+02	5.8	0.93	9.0E-03	9.0E-04	4.8E-02
Concert hall/theater	85	2.4	1	2	300	1.3E+04	0.49	0.36	7.0E+00	2.3E-02	3.5E+01
	85	2.4	0.35	1	150	1.3E+04	3.5	0.72	1.7E-01	1.1E-03	9.2E-01

Restaurant	4.7	1	1	1	50	2.1E+02	4.3	0.77	2.0E-01	3.9E-03	1.0E+00
	2.9[b]	1	1[c]	1[d]	25	2.1E+02	7.3	0.86	4.0E-02	1.6E-03	2.1E-01
Hotel lobbies/prefunction	2.8	2.4	1	8	50	1.0E+03	0.86	0.86	2.7E+00	5.3E-02	1.2E+01
	2.8	2.4	0.35	4	25	1.0E+03	3.9	0.94	5.7E-02	2.3E-03	3.0E-01
Airport terminal /railway station	2.8	2.4	1	1	1000	1.0E+04	1.5	0.48	2.2E-01	2.2E-04	1.2E+00
	2.8	2.4	0.35	1[d]	500	1.0E+04	4.5	0.78	2.0E-02	4.1E-05	1.1E-01
Hospital general examination room	50[e]	1	0.35[f]	8	20	3.0E+02	1.6	0.92	5.3E+00	2.6E-01	1.5E+01
	50[e]	1	0.01[g]	4	10	3.0E+02	9.0[h]	0.97	7.2E-03	7.2E-04	3.9E-02
Library	1	1	1	2	100	3.0E+03	1.0	0.57	3.7E-02	3.7E-04	2.0E-01
	1	1	0.35	2[d]	50	3.0E+03	4.0	0.88	2.5E-03	5.1E-05	1.4E-02
Museum/gallery	1.2	1.5	1	2	200	5.0E+03	0.66	0.44	9.7E-02	4.9E-04	5.2E-01
	1.2	1.5	0.35	2[d]	100	5.0E+03	3.7	0.86	6.0E-03	6.0E-05	3.2E-02
Place of religious worship	30	1	1	2	100	8.3E+02	1.2	0.62	3.8E+00	3.8E-02	1.8E+01
	4.7[i]	1	0.35	1	50	8.3E+02	4.2	0.76	1.8E-02	3.6E-04	9.6E-02
Mall common area	2.8	2.4	1	2	500	7.5E+03	1.1	0.59	4.9E-01	9.7E-04	2.6E+00
	2.8	2.4	0.35	1	250	7.5E+03	4.1	0.76	1.5E-02	5.8E-05	7.8E-02

Supermarket	2.8	2.4	1	8	100	7.5E+03	0.36	0.67	1.3E+00	1.3E-02	6.9E+00
	2.8	2.4	0.35	4	50	7.5E+03	3.4	0.93	1.7E-02	3.5E-04	9.2E-02
Gym, sports arena (play area)	6.8	9	1	1	100	1.4E+04	0.58	0.24	1.8E-01	1.8E-03	9.5E-01
	6.8	9	0.35	0.5	50	1.4E+04	3.6	0.53	5.6E-03	1.1E-04	3.0E-02
Other cases											
Physical education class	6.8	9	1	1	30	1.3E+03	1.9	0.56	4.1E-01	1.4E-02	2.1E+00
	6.8	9	0.35	0.5	15	1.4E+04[j]	4.9	0.63	1.4E-03	9.6E-05	7.7E-03
Subway car[k]	1	1	1	0.33	30	1.5E+02	5.7	0.55	6.5E-03	2.2E-04	3.5E-02
	1	1	0.35	0.33[d]	30[l]	1.5E+02	9.3[m]	0.69	1.7E-03	5.8E-05	9.3E-03
Large family dinner	4.7	1	1	2	12	3.0E+02	0.5	0.37	2.8E-01	2.3E-02	1.4E+00
	2.9[b]	1	0.35	2[d]	6	3.0E+02	3.5	0.86	9.9E-03	1.7E-03	5.3E-02
Shared office	4.7	1	1	8	2	3.4E+01	2	0.94	1.0E+00	5.2E-01	1.9E+00
	1.7[n]	1	0.35	4	1	3.4E+01	5	0.95	1.3E-02	1.3E-02	6.9E-02
Large university classroom[o]	30	1	1	1	150	7.0E+02	2	0.57	1.8E+00	1.2E-02	9.5E+00
	4.7[i]	1	0.35	1[c]	60[p]	7.0E+02	11[q]	0.91	1.2E-02	1.9E-04	6.2E-02
University laboratory	2.8	2.4	1	8	10	2.3E+02	6	0.98	3.9E-01	3.9E-02	1.9E+00
	2.8	2.4	0.35	4	3[o]	2.3E+02	9	0.97	1.3E-02	4.5E-03	7.1E-02

Outbreaks											
Guangzhou restaurant	9.3[r]	1	1	1.2	20	9.7E+01	0.67	0.31	1.1E+00	5.4E-02	5.0E+00
	4.7[s]	1	1[c]	0.6	10	9.7E+01	3.67	0.60	4.7E-02	4.7E-03	2.5E-01
Skagit Choir	85	2.5[t]	1	2.5	60	8.1E+02	0.7	0.53	3.0E+01	5.0E-01	5.6E+01
	85	2.5[t]	0.35	1.3	30	8.1E+02	3.7	0.79	7.3E-01	2.4E-02	3.7E+00

Footnotes: [a] for sedentary teenagers; [b] half resting - oral breathing + half resting - speaking; [c] no face covering; [d] no duration reduction; [e] for a coughing infector (see Footnote e of Table 1 for detail of the estimation); [f] use of surgical masks for the pre-pandemic setting; [g] N95 respirators and fit tests required (resulting in f_e and f_i of 0.1) before allowed indoors; [h] ventilation rate increased to 6 h^{-1}; [i] reduction of vocalization level from loudly speaking to speaking (with the aid of, for example, microphone); [j] use of a much larger room if the event has to be indoors; [k] real-world case;[4] [l] no occupancy reduction; [m] λ_{cle} = 3.6 h^{-1}; [n] 4/5 resting - oral breathing + 1/5 resting - speaking; [o] real-world case;[10] [p] occupancy reduction larger than 50%; [q] ventilation rate increased to the maximum and no additional virus removal applied; [r] talking during half of the time and half normal / half loud talking assumed; [s] resting - speaking; [t] light intensity for 61-<71 years.

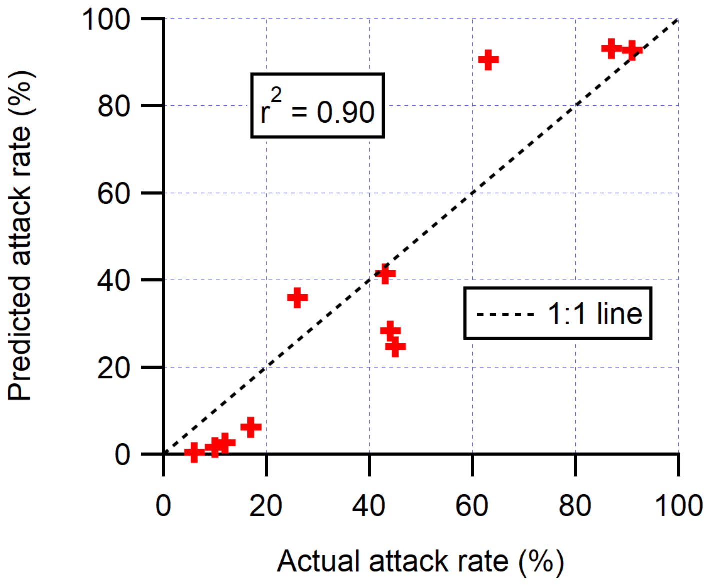

Figure S1: Attack rates of the COVID-19 outbreaks shown in Table 1 predicted according to the fitted trend line in Figure 1b vs. actual attack rates of those outbreaks. The correlation coefficient between the two types of attack rates and the 1:1 line are also shown.

	A	B	C	D	E	F	G	H
1								
2	**Estimation of COVID-19 aerosol transmission: master spreadsheet, adapt this one to your case - Default values are for Skagit Choir outbreak**							
3								
4	This is a general spreadsheet applicable to any situation, under the assumptions of this model - *See notes specific to this case (if applicable) at the very bottom*							
5	Important inputs as highlighted in orange - change these for your situation							
6	Other, more specialized inputs are highlighted in yellow - change only for more advanced applications							
7	Calculations are not highlighted - don't change these unless you are sure you know what you are doing							
8	Results are in blue -- these are the numbers of interest for most people							
9								
10	**Environmental Parameters**							
11								
12		**Value**			**Value in other units**		**Source / Comments**	
13	Length of room	30	ft		9.2	m	Can enter as ft or as m (once entered as m, changing in ft does not work)	
14	Width of room	60	ft	=	18.3	m	Can enter as ft or as m (once entered as m, changing in ft does not work)	
15		1800	sq ft		167	m2	Can overwrite the m2 one. If you want to enter sq ft, enter "=B15*0.305^2" in the m2 cell, where B15 is the cell w/ sq f	
16	Height	16	ft	=	4.8	m	Can enter as ft or as m (once entered as m, changing in ft does not work)	
17	Volume				810	m3	Volume, calculated. (Can also enter directly, then changing dimensions does not work)	
18								
19	Pressure	0.95	atm				Used only for CO2 calculation	
20	Temperature	20	C				Use **web converter** if needed for F --> C. Used for CO2 calculation, eventually for survival rate of virus	
21	Relative Humidity	50	%				Not yet used, but may eventually be used for survival rate of virus	
22	Background CO2 Outdoors	415	ppm				See readme	
23								
24	Duration of event	150	min		2.5	h	Value for your situation of interest	
25								
26	Number of repetitions of event	1	times				For e.g. multiple class meetings, multiple commutes in public transportation etc.	
27								
28	Ventilation w/ outside air	0.7	h-1				Value in h-1: Readme: Same as "air changes per hour". Value in L/s/per to compare to guidelines (e.g. ASHRAE 62.1	
29	Decay rate of the virus	0.62	h-1				See Readme, can estimate for a given T, RH, UV from DHS estimator	
30	Deposition to surfaces	0.3	h-1				Buonnano et al. (2020), Miller et al. (2020). Could vary 0.24-1.5 h-1, depending on particle size range	
31	Additional control measures	0	h-1				E.g. filtering of recirc. air, HEPA air cleaner, UV disinfection, etc. See FAQs, Readme for calc for portable HEPA filter	
32	Total first order loss rate	1.62	h-1				Sum of all the first-order rates	
33								
34	Ventilation rate per person	2.6	L/s/person				This is the value of ventilation that really matters for disease transmission. Includes additional control measures	

+ Readme | FAQs | Master-Choir | Class | Subway | Supermkt | Stadium | Class_Old | Outdoors_Old | Campus_Old | Bus_Old

Figure S2: screenshot of the COVID-19 Aerosol Transmission Estimator.[11] The top of the sheet simulating the Skagit Valley choir outbreak is shown.

S1. Deviation of quanta concentration from steady state due to finite event duration

In case of short events where quanta concentration does not reach steady state, a correction factor, r_{ss}, can be introduced to account for the deviation of average quanta concentration (c_{avg}) from that at steady state (c):

$$r_{ss} = c_{avg} / c \qquad \text{(S1)}$$

Under the assumption of no infectious quanta in the air at the beginning of the event, c_{avg} can be easily obtained from the integration of equation (1). Details of the derivation can be found elsewhere.[4,9] For a period [0, D],

$$c_{avg} = E_p\ f_e / (V\ \lambda) \times (1 - (1 - e^{-\lambda D}) / (\lambda\ D)) \qquad \text{(S2)}$$

Inserting equations (2) and (S2) into equations (S1) yields:

$$r_{ss} = 1 - (1 - e^{-\lambda D}) / (\lambda\ D) \qquad \text{(S3)}$$

The value of r_{ss} as a function of λD is shown in Figure S3. r_{ss} approaches to λD/2 when λD is very small and to 1 when λD is very large, and reaches 0.6 at λD ~ 2.

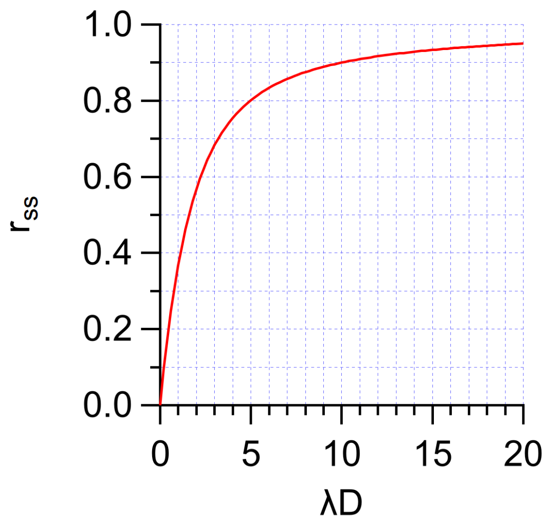

Figure S3: Ratio of the quanta concentration averaged over a period [0, D] to that at steady state (r_{ss}) as a function of the product of total first-order quanta loss rate constant (λ) and the event duration (D).

When D is very small (e.g., a few minutes or shorter), it may be too short for the air in the space of interest to become well mixed. In this case, the box modeling approach is no longer suitable. Nevertheless, whether D is too short also depends on the internal mixing rate of the air in the space of interest. When the latter is enhanced (e.g., by fans), the well-mixing assumption can still hold even with a small D.

S2. Monte Carlo uncertainty propagation for the fitting of attack rates vs. H_r

We follow the standard procedure of Monte Carlo uncertainty propagation[12] for the fitting of attack rates vs. H_r. We assume log-normal distributions for the variables constraining H_r (r_E, r_B, D, V, and λ; f_e and f_i are excluded as little to no mask wearing was reported for the COVID-19 outbreaks analyzed in this study) to ensure positive values of their samples. r_E, r_B, D, V, and λ are assigned uncertainty factors of 2.5, 1.3, 1.1, 1.3, and 1.9 respectively (approximately corresponding to relative uncertainties of 150%, 30%, 10%, 30%, and 90%). The last three uncertainties are typical values for outbreak case studies. The uncertainty factor of 1.3 for r_B mainly reflects the possible error arising from the discretization of physical intensity levels in the [3] dataset. We assume an uncertainty factor of 2.5 for r_E because Buonanno et al.[1] estimated the uncertainty of $E_{p0}xr_E$ for COVID-19 to be an order of magnitude and we think that r_E, a relative factor that depends largely on type of activity but not on that of disease, contributes only a minority of this uncertainty. Since attack rate (AR) is bounded between 0 and 100%, it does not follow a log-normal distribution. We use a similar transformation as in Gans et al.[13], i.e., AR / (1 - AR), to expand the domain of the samples from [0, 1] to [0, +∞). The intermediate samples then can be depicted with a log-normal distribution. We assign an uncertainty factor of 1.1 to the intermediate samples. The generated samples are then reversely transformed into the AR samples. When AR is small, the assigned uncertainty factor of 1.1 approximately corresponds to

a relative uncertainty of 10% for AR; while when AR is close to 1, this uncertainty factor reflects an approximate relative uncertainty of 10% for non-attack rate, i.e., (1 - AR). 10000 random samples of r_E, r_B, D, V, λ, and AR are generated for each of the COVID-19 case studies in Table 1. A fitting can be done for one sample of r_E, r_B, D, V, λ, and AR of all those case studies, yielding a sample of the fitted parameter, E_{p0}. This fitting is repeated for all 10000 samples of the input parameters, giving 10000 samples of E_{p0}, apparently log-normally distributed, with 5th and 95th percentiles being 8.4 and 48.1 quanta h^{-1}, respectively.

References

(1) Buonanno, G.; Stabile, L.; Morawska, L. Estimation of Airborne Viral Emission: Quanta Emission Rate of SARS-CoV-2 for Infection Risk Assessment. *Environ. Int.* **2020**, *141* (April), 105794.

(2) Buonanno, G.; Morawska, L.; Stabile, L. Quantitative Assessment of the Risk of Airborne Transmission of SARS-CoV-2 Infection: Prospective and Retrospective Applications. *Environ. Int.* **2020**, *145*, 106112.

(3) EPA. Chapter 6—Inhalation Rates. In *Exposure Factors Handbook*; U.S. Environmental Protection Agency, 2011.

(4) Peng, Z.; Jimenez, J. L. Exhaled CO2 as a COVID-19 Infection Risk Proxy for Different Indoor Environments and Activities. *Environmental Science & Technology Letters* **2021**. https://doi.org/10.1021/acs.estlett.1c00183.

(5) Klink, K. Trends in Mean Monthly Maximum and Minimum Surface Wind Speeds in the Coterminous United States, 1961 to 1990. *Clim. Res.* **1999**, *13*, 193–205.

(6) ASHRAE. *Ventilation for Acceptable Indoor Air Quality: ANSI/ASHRAE Standard 62.1-2019*; ANSI/ASHRAE, 2019.

(7) Davies, A.; Thompson, K.-A.; Giri, K.; Kafatos, G.; Walker, J.; Bennett, A. Testing the Efficacy of Homemade Masks: Would They Protect in an Influenza Pandemic? *Disaster Med. Public Health Prep.* **2013**, *7* (4), 413–418.

(8) Li, Y.; Qian, H.; Hang, J.; Chen, X.; Cheng, P.; Ling, H.; Wang, S.; Liang, P.; Li, J.; Xiao, S.; Wei, J.; Liu, L.; Cowling, B. J.; Kang, M. Probable Airborne Transmission of SARS-CoV-2 in a Poorly Ventilated Restaurant. *Build. Environ.* **2021**, *196*, 107788.

(9) Miller, S. L.; Nazaroff, W. W.; Jimenez, J. L.; Boerstra, A.; Buonanno, G.; Dancer, S. J.; Kurnitski, J.; Marr, L. C.; Morawska, L.; Noakes, C. Transmission of SARS-CoV-2 by Inhalation of Respiratory Aerosol in the Skagit Valley Chorale Superspreading Event. *Indoor Air* **2021**, *31* (2), 314–323.

(10) Liu, S.; Li, R.; Wild, R. J.; Warneke, C.; de Gouw, J. A.; Brown, S. S.; Miller, S. L.; Luongo, J. C.; Jimenez, J. L.; Ziemann, P. J. Contribution of Human-Related Sources to

Indoor Volatile Organic Compounds in a University Classroom. *Indoor Air* **2016**, *26* (6), 925–938.
(11) Jimenez, J. L.; Peng, Z. COVID-19 Aerosol Transmission Estimator https://tinyurl.com/covid-estimator (accessed Mar 26, 2021).
(12) Bipm; Iec; Ifcc; Ilac; Iso; Iupac; Iupapoiml. *JCGM 101: 2008 Evaluation of Measurement Data — Supplement 1 to the “ Guide to the Expression of Uncertainty in Measurement ” — Propagation of Distributions Using a Monte Carlo Method*; 2008.
(13) Gans, B.; Peng, Z.; Carrasco, N.; Gauyacq, D.; Lebonnois, S.; Pernot, P. Impact of a New Wavelength-Dependent Representation of Methane Photolysis Branching Ratios on the Modeling of Titan’s Atmospheric Photochemistry. *Icarus* **2013**, *223* (1), 330–343.
